# Supplementary figures and images for: Age and the association between apolipoprotein E genotype and Alzheimer disease: A cerebrospinal fluid biomarker–based case–control study
Source: PLoS Med. 2020 Aug 20;17(8):e1003289. doi: 10.1371/journal.pmed.1003289 (PMC7446786; doi:10.1371/journal.pmed.1003289)

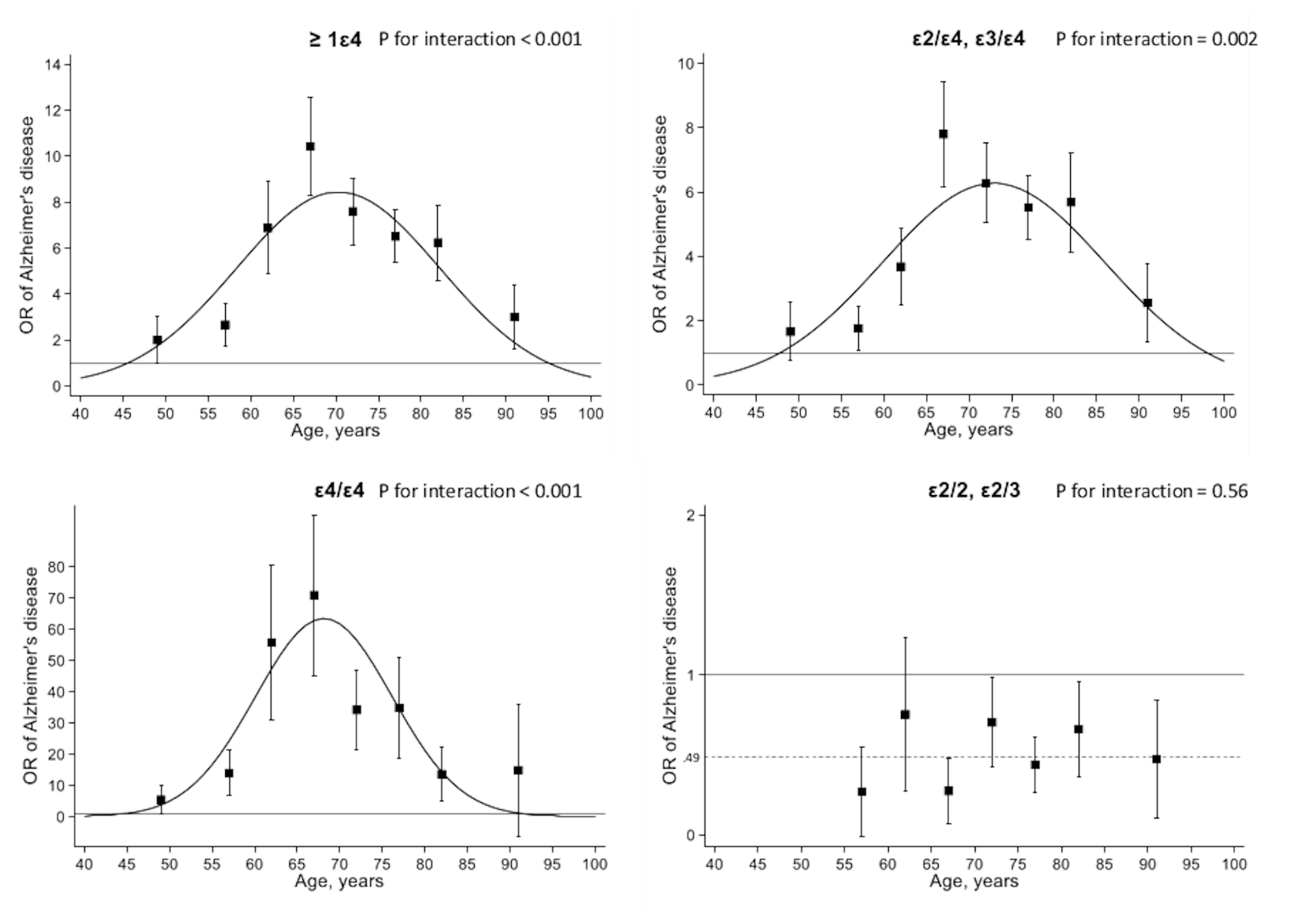

Supplement: S1 Fig — Genotype ε3/ε3 was used as reference. Associations between age and OR of AD were modeled using a quadratic term for age in the logistic regression model and adjusted for sex, education, hypertension, diabetes mellitus, and hypercholesterolemia. AD, Alzheimer disease; APOE, apolipoprotein E; CSF, cerebrospinal fluid; OR, odds ratio. (TIF) [file pmed.1003289.s002.tif]
