# Supplementary material for: Age and the association between apolipoprotein E genotype and Alzheimer disease: A cerebrospinal fluid biomarker–based case–control study
Source: PLoS Med. 2020 Aug 20;17(8):e1003289. doi: 10.1371/journal.pmed.1003289 (PMC7446786; doi:10.1371/journal.pmed.1003289)
Supplement: S1 Table — Aβ, β-amyloid; CSF, cerebrospinal fluid; p-Tau 181, tau phosphorylated at threonine 181. (DOCX) [file pmed.1003289.s003.docx]

**Supplementary Table 1.** Thresholds levels used for defining abnormal values of CSF Aβ42, CSF Tau and CSF p-Tau 181 in the memory centers that participated to the study.

|  |  |  |  |  |  |  |
| --- | --- | --- | --- | --- | --- | --- |
|  | Number of | |  | CSF biomarkers (pg/mL) cut-offs | | |
| Centers | CSF AD | CSF controls |  | Aβ42 | Tau | p-Tau 181 |
| Paris | 439 | 316 |  | 500 | 300 | 58 |
| Rouen | 64 | 0 |  | 700 | 400 | 60 |
| Montpellier | 36 | 66 |  | 500 | 350 | 60 |
| Gothenburg | 159 | 101 |  | 450 | 400 | — |
| Barcelona | 32 | 56 |  | 550 | 350 | 61 |
| Munich | 58 | 2 |  | 642 | 252 | 61 |
| Perugia | 51 | 0 |  | 500 | 400 | 64 |
| Antwerp | 227 | 101 |  | 638 | 296 | 56 |
| Göttingen | 49 | 8 |  | 450 | 450 | 61 |
| ADNI Study | 478 | 155 |  | 192 | 93 | 23 |
|  |  |  |  |  |  |  |
