# Supplementary material for: Age and the association between apolipoprotein E genotype and Alzheimer disease: A cerebrospinal fluid biomarker–based case–control study
Source: PLoS Med. 2020 Aug 20;17(8):e1003289. doi: 10.1371/journal.pmed.1003289 (PMC7446786; doi:10.1371/journal.pmed.1003289)
Supplement: S2 Table — AD, Alzheimer disease; CSF, cerebrospinal fluid. (DOCX) [file pmed.1003289.s004.docx]

**Supplementary table 2.** The Odds ratios of AD in CSF AD cases compared to population controls, before and after exclusion of the center with missing CSF phosphorylated tau.

|  |  |  |  |  |  |
| --- | --- | --- | --- | --- | --- |
|  | All centers | |  | Without Gothenburg | |
| *APOE* genotype | OR (95% CI) | p-value |  | OR (95% CI) | p-value |
| 0 ε4 | 1 (Ref) | . |  | 1 (Ref) | . |
| ≥ 1 ε4 | 5.9 (5.3 - 6.6) | <0.001 |  | 5.6 (5.0 - 6.3) | <0.001 |
|  |  |  |  |  |  |
| 0 ε4 | 1 (Ref) | . |  | 1 (Ref) | . |
| 1 ε4 | 4.6 (4.1 - 5.2) | <0.001 |  | 4.5 (3.9 - 5.0) | <0.001 |
| 2 ε4 | 25.4 (20.4 - 31.2) | <0.001 |  | 23.6 (19.0 - 29.4) | <0.001 |
|  |  |  |  |  |  |
| ε2/ε2, ε2/ε3 | 0.68 (0.53 - 0.88) | 0.003 |  | 0.62 (0.47 - 0.82) | 0.001 |
| ε3/ε3 | 1 (Ref) | . |  | 1 (Ref) | . |
| ε2/ε4 | 2.9 (2.0 - 4.0) | <0.001 |  | 2.6 (1.8 - 3.7) | <0.001 |
| ε3/ε4 | 4.5 (4.0 - 5.1) | <0.001 |  | 4.3 (3.8 - 4.9) | <0.001 |
| ε4/ε4 | 23.9 (19.3 - 29.6) | <0.001 |  | 22.1 (17.7 - 27.6) | <0.001 |
| Abbreviations: AD. Alzheimer’s disease; *APOE*. Apolipoprotein E; CSF. Cerebrospinal Fluid; OR. odds ratio; CI. confidence intervals. | | | | | |
