# Supplementary material for: Age and the association between apolipoprotein E genotype and Alzheimer disease: A cerebrospinal fluid biomarker–based case–control study
Source: PLoS Med. 2020 Aug 20;17(8):e1003289. doi: 10.1371/journal.pmed.1003289 (PMC7446786; doi:10.1371/journal.pmed.1003289)
Supplement: S3 Table — AD, Alzheimer disease; APOE, apolipoprotein E. (DOCX) [file pmed.1003289.s005.docx]

**Supplementary Table 3.** Characteristics of the sub-sample used for the assessment of the association between *APOE* genotype and AD adjusted for cardiovascular risk factors.

|  |  |  |  |
| --- | --- | --- | --- |
|  | CSF AD | Population controls |  |
| Characteristics | (N=841) | (N=11,665) | P-value |
| Age, years, mean (SD) | 72.9 (7.9) | 65.6 (10.7) | <0.001 |
| Women, n (%) | 441 (52.4) | 5491 (47.1) | 0.003 |
| MMSE, mean (SD) | 21.8 (5.8) | 27.5 (1.8) | <0.001 |
| Education, n (%) |  |  | <0.001 |
| Low | 66 (7.8) | 610 (5.2) |  |
| Medium | 201 (23.9) | 9033 (77.4) |  |
| High | 574 (68.3) | 2022 (17.3) |  |
| *APOE* genotype, n (%) |  |  | <0.001 |
| ε2/ε2 | 2 (0.2) | 72 (0.6) |  |
| ε2/ε3 | 29 (3.4) | 1437 (12.3) |  |
| ε3/ε3 | 279 (33.2) | 7518 (64.4) |  |
| ε2/ε4 | 20 (2.4) | 220 (1.9) |  |
| ε3/ε4 | 376 (44.7) | 2258 (19.4) |  |
| ε4/ε4 | 135 (16.1) | 160 (1.4) |  |
| Hypertension, n (%)^a^ | 412 (49.0) | 5249 (45.0) | 0.024 |
| Diabetes, n (%)^b^ | 122 (14.5) | 616 (5.3) | <0.001 |
| Hypercholesterolemia, n (%)^c^ | 416 (49.5) | 4644 (39.8) | <0.001 |
| Abbreviations : AD, Alzheimer’s disease; *APOE*, Apolipoprotein E; CSF, Cerebrospinal Fluid; | | | |
| SD, standard deviation. | | | |
| ^a^ Systolic/diastolic blood pressure ≥ 140/90 mmHg or use of antihypertensive treatment. | | | |
| ^b^ Fast glycemia ≥ 1.26 g/L or use of antidiabetics drugs. | | | |
| ^c^ LDL cholesterol ≥ 1.9 g/L or use of lipid-lowering treatment. | | | |
