# Supplementary material for: Age and the association between apolipoprotein E genotype and Alzheimer disease: A cerebrospinal fluid biomarker–based case–control study
Source: PLoS Med. 2020 Aug 20;17(8):e1003289. doi: 10.1371/journal.pmed.1003289 (PMC7446786; doi:10.1371/journal.pmed.1003289)
Supplement: S1 Text — (DOCX) [file pmed.1003289.s006.docx]

**English translation of the proposed MSc internship project**

This project was posted on the ISPED website (http://www.isped.u-bordeaux.fr/ISPED/On-recrute), Institut de santé publique, d'épidémiologie et de développement, University of Bordeaux, France, on November 11^th^ 2018 (N° 2018-5380).

**Title of the subject:**
Association between APOE4 and Alzheimer's disease with positive CSF biomarkers.
 
 
**Research question:**
What is the association between APOE4 genotype and Alzheimer's disease, when Alzheimer's disease is defined on the positivity of cerebrospinal fluid tau and beta-amyloid biomarkers using A/T/N classification ?
 
  
**Population studied :**
Case: Alzheimer patients with positive biomarkers from several European and American memory clinics : A+T+.
Controls: drawn from 2 longitudinal cohorts in general population: Three-City and Whitehall-II studies.
 
**Data source:**
European memory clinics: France (Paris, Rouen, Montpellier), Sweden (Gothenburg), Spain (Barcelona), Germany (Munich, Göttingen), Italia (Perugia), and Belgium( Antwerp).
ADNI Study.
Whitehall II Study.
3-City Study.

**Variables to be analyzed:** 
- APOE genotyping.
- Biomarkers of cerebrospinal fluid: beta-amyloid, total tau, phosphorylated tau.
- A/T/N classification.
- Age.
- Sex.
- Level of education.
 
**Statistical analysis strategy:**
- Prevalence of APOE4 by age-group in AD cases and controls.
- Definition of 2 kind of controls: general population after exclusion of incident dementia (Whitehall II, Three-City), and patients form the same memory clinics than AD, defined by normal values for CSF biomarkers (A-T-N-).
- Description of the age of onset among AD cases according to APOE4 status.
- Overall and age-specific odds ratio of AD according to APOE4 status using logistic regression models. Quadratic modelling of age (age and age*age).
- Interaction of sex and level of education on the relationship between APOE4 and age, and stratified analysis.
