# Supplementary material for: Age and the association between apolipoprotein E genotype and Alzheimer disease: A cerebrospinal fluid biomarker–based case–control study
Source: PLoS Med. 2020 Aug 20;17(8):e1003289. doi: 10.1371/journal.pmed.1003289 (PMC7446786; doi:10.1371/journal.pmed.1003289)
Supplement: Attachment — Submitted filename: Response to reviewers.docx [file pmed.1003289.s007.docx]

**EDITORS**

**1.** Please adapt the title so that it is not declarative. We suggest "Age and the association between apolipoprotein E genotype and Alzheimer’s disease: a cerebrospinal fluid biomarker-based case-control study.

**OUR RESPONSE:** Thank you for this suggestion. We have changed the title as follows (page 1, lines 1-2):

*“Age and the association between apolipoprotein E genotype and Alzheimer’s disease: a cerebrospinal fluid biomarker-based case-control study.”*

**2**. In the abstract and elsewhere, please add p values alongside CI where available.
**OUR RESPONSE:** As requested, p values are now provided alongside 95% CI in the manuscript**.**

**3.** Please add summary demographic details for study participants to the abstract.
**OUR RESPONSE:** Thank you, we have added summary demographic information on participants in the revised manuscript as follows:

*“This case-control study include: 1,593 Caucasian AD cases (55.4% women; mean age 72.8 (range=44-96) years) with abnormal values of CSF biomarkers came from 9 European memory clinics and the ADNI study; and 11,723 dementia-free controls (47.1% women; mean age 65.6 (range=44-94) years), drawn from two longitudinal cohort studies (Whitehall II and 3-City).”* (page 3, line 73-75)

**4.** Please add a new final sentence to the "methods and findings" subsection of your abstract, which should summarize the study's main limitations.
**OUR RESPONSE:** We have added details of the main limitations of the study as follows:

“Main study limitations are that analyses were based on Caucasian subjects and AD cases were drawn from memory centers which may not be representative of the general population of AD patients.” (page 4, lines 87-89)

**5.** Please begin the sentence at line 86 with "In this study, we found that ..." or similar.
**OUR RESPONSE:** Thank you. As suggested, the conclusion now reads as follows:

*“In this study, we found that AD has a stronger association with APOE ε4 than previously reported when AD diagnosis is based on biomarkers.”*(page 4, line 91)

**6.** After the abstract, we will need to ask you to add a new and accessible "author summary" section in non-identical prose. You may find it helpful to consult one or two recent research papers published in PLOS Medicine to get a sense of the preferred style.

**OUR RESPONSE:** We have added the author summary after the abstract, see page 5-6.

**7.** Early in the methods section of your main text, please state whether the present study had a protocol or prespecified analysis plan, and if so attach the relevant document(s) as an attachment, referred to in the text. Please highlight analyses that were not prespecified.

**OUR RESPONSE:** The analyses undertaken were proposed as an MSc internship project advertised via various university channels in November 2018. The first author, Hana Saddiki, from University of Caen was chosen by the team to undertake this project. We now provide a translation from French of the advertised project and refer to this document in the second sentence of the Methods section as follows (page 7, lines 166-170):

*“The study objectives and analysis plan were developed prior to data manipulation as an MSc internship project (S1 Text). The analyses undertaken in response to reviewers’ suggestions are presented in post-hoc analyses*.*”*

**8.** At line 345 and any other instances, please avoid claims of "the first" or similar, and where these are needed please add "to our knowledge", for example.

**OUR RESPONSE:** We have modified this sentence accordingly (page 25, line 432-434):

*“Our use of large, multicentric study of AD patients defined by the new A/T/N criteria allows the risk of misclassification bias to be addressed as it can be critical in such analysis.”*

**9.** Where study limitations are summarized (at line 412), we suggest crafting a dedicated paragraph and providing a fuller discussion.

**OUR RESPONSE:** As suggested, we have added a dedicated paragraph for “Strengths and Limitations” and provided a fuller discussion (page 27-28, lines 488-514):

**10.** In the reference list, please adapt citations so that, where appropriate, six rather than five authors' names are listed, followed by "et al.".

**OUR RESPONSE:** As requested, we have revised the citations (page 30-34).

**11.** For reference 21, please make the journal name "BMJ".

**OUR RESPONSE:** This has been corrected (reference 25).

**12.** Please adapt the attached STROBE checklist so that individual items are referred to by section (e.g., "Methods") and paragraph number rather than by page or line numbers, as the latter generally change in the event of publication. Please refer to this in the methods section of your main text.

**OUR RESPONSE:** We have revised the STROBE checklist to match the paragraph numbers and sections from the manuscript rather than page or line numbers.

**REVIEWER 1**

Thank you for the opportunity to review this very interesting and compelling study by Saddikki and colleagues on exploring the association between the APOE e genotype and dementia.  This retrospective study used brought together a consortia of AD patients and non-AD controls from Whitehall II 3-City to determine the effect size of association between APOE e4 allele (one/two copies) and AD, provide PAFs by age-stratum. The author did find an significant association (with stronger effects seen in the homozygotes) with the effect modified by age. The effect sizes were much higher than previously reported in published evidence, however it has been well-established from previous meta-analysis and case-control studies that APOE genotypes are associated strongly with AD. The case control design is an appropriate design for this analysis and the statistical analysis was simple and straightforward.

However, my major comments regards on controls selection, imbalance between cases and controls which will require some form of covariate adjustment or propensity score matching as the effect sizes of unadjusted ORs may be overestimated. Specific comments related to this:

**1.** The authors utilized two large community dwelling cohorts to source controls from in the UK and France with APOE genotyping with 11,724 controls. The ratio between AD cases and controls is therefore approx. 1:7. Previous evidence has suggested that ORs for APOE genotypes are order of magnitude of > 3. I'm surprised the authors did not report a sample size calculation based on expected detection to get a sense of the minimum ratio of controls to cases. I mention this because it's likely the study is overpowered. While larger number of controls may seem obviously better, there may have been an opportunity to do a more sophisticated selection technique to account for selection and indication bias (which the authors themselves discuss as a limitation) given set a large number of controls. Authors clarification and comment on this aspect would be useful here.

**OUR RESPONSE:** Thank you for raising this interesting issue. A study being overpowered is mostly a concern when large sample sizes are used to detect small “statistically significant” differences. This is not the case for *APOE* and AD where the association is robust. For a 25% prevalence of *APOE* ε4 carriers in controls, and a presumed OR of 3 for the association with AD, 55 participants per group would be enough (power 80% at p<0.05). However, our analysis goes further by considering the effect of the detailed *APOE* genotypes using 5 years age categories (8 age-groups). Furthermore, the association between *APOE* and AD appears to be weaker in both tails of distribution of age. The AD group < 55 years contains only 43 patients. The ε2/ε2 genotype was rare, even with 11,000 controls group it contained only 73 (0.6%) individuals. While 4 or 5 controls per case is considered to be sufficient in case-control studies, an higher case-control ratio is desirable when the cost of including additional controls is negligible and when sufficient numbers are needed for stratified analyses [1] as is our case.

We have added the following discussion to the “Strengths and Limitations” section of the revised manuscript (page 28 , lines 502-514):

*“Third, the overall ratio between AD cases and controls used in this study was around 1 to 7, which may lead to detection of non-pertinent associations due to an overpowered design. However, the value of a large sample size is the ability to examine the association between APOE and AD in age sub-groups to show the manner in which age modifies this association [43].”*

**2.** Related the above comment then, I brings up the control selection because in Table 1, there are significant imbalances in age, gender, MMSE, education, CSF biomarkers (P < 0.001) and these are just the covariates that were reported. I would venture to guess that there are quite  few more covariates captured. In the subsequent analysis of the logistic regression models (Table 2), there was no indication any covariate adjustments were made. In fact, there seems to be no consideration of prior history vascular conditions (for instance APOE genotypes are associated with familial hypercholesterolaemia and atherosclerosis - [https://clicktime.symantec.com/3JK7nx8gsW3oBBGGg7wZjid6H2?u=https%3A%2F%2Fwww.ncbi.nlm.nih.gov%2Fpmc%2Farticles%2FPMC5505295%2F](https://courriel-bsr.aphp.fr/owa/redir.aspx?C=bO2MWumZ8It6HU6JrOk0Hlun6tEAtKoHdul0RREgnrdUGSxSTbXXCA..&URL=https%3a%2f%2fclicktime.symantec.com%2f3JK7nx8gsW3oBBGGg7wZjid6H2%3fu%3dhttps%253A%252F%252Fwww.ncbi.nlm.nih.gov%252Fpmc%252Farticles%252FPMC5505295%252F)). Reporting crude ORs is good but what would improve the analysis and be more convincing if adjusted analyses could also be provided to account for confounders. One method that could help deal with residual confounding is a the use of propensity scores adjustment ([https://clicktime.symantec.com/3HbV6fHuafX8SF6Hot4zqs46H2?u=https%3A%2F%2Fwww.sciencedirect.com%2Fscience%2Farticle%2Fpii%2FS1476927116300135](https://courriel-bsr.aphp.fr/owa/redir.aspx?C=ihdqWf3s8sLUTMFyQa2gMwlpppzJL5HYLR0KpzDqx55UGSxSTbXXCA..&URL=https%3a%2f%2fclicktime.symantec.com%2f3HbV6fHuafX8SF6Hot4zqs46H2%3fu%3dhttps%253A%252F%252Fwww.sciencedirect.com%252Fscience%252Farticle%252Fpii%252FS1476927116300135)) - creating a regression model (based on distribution of all the covariates) and then using the score to adjust during the model. This does not necessarily need to be done if simple covariate adjustment, considering confounding factors such as vascular comorbidities was taken into account. Barring that, covariate adjustment in adjusted model should be considered.

**OUR RESPONSE:** We thank the reviewer for raising this interesting point. Most previous case-controls studies report the unadjusted OR of the association between *APOE* and AD. Our objective was to show that this approach misses the fact that this association is strongly dependent of age. Use of a single overall OR may not be suitable to describe the association. As suggested by the reviewer, we undertook additional analysis to investigate the effect of sex, education, and key cardiovascular risk factors (hypertension, diabetes, hypercholesterolemia) on a subsample of 841 CSF AD cases for whom these data were available. There were no significant differences in term of age (p=0.80), and APOE ε4 distribution (p=0.67) between included and non-included CSF AD patients. Compared to population controls, CSF AD cases were more likely to have hypertension, diabetes mellitus, and hypercholesterolemia, these results are included as Supplementary Table 3 in the revised manuscript.

We then examined the effect of adjustment for sex, education, hypertension, diabetes mellitus, and hypercholesterolemia using multivariable logistic regression analyses, presented as Table 3 in the revised manuscript. Supplementary Figure 1 shows this multivariable model (age, sex, education, hypertension, diabetes mellitus, and hypercholesterolemia) stratified by age-group. Additional adjustments on these covariates had little impact on the estimates of ORs compared to the unadjusted model. These results are discussed in the revised manuscript as follows:

- Page 23, line 404-414:
  *“The final analysis was on the subset of AD patients (841 CSF AD cases) with data on cardiovascular risk factors. There were no significant differences between CSF AD cases with and without these data in term of age (p=0.80) and APOE genotype (p=0.67). Compared to general population controls (supplementary table 3), CSF AD cases were more likely to have hypertension (p=0.024), diabetes mellitus (p<0.001), and hypercholesterolemia (p<0.001). We examined the role of these risk factors in the association of APOE genotypes with AD without adjustment for age due to its role in modifying the association between APOE genotype and AD. Table 3 shows these results. Adjustment for sex and education (model 1), and for cardiovascular risk factors (model 2) had little impact on the estimates compared to the unadjusted model. Supplementary Figure 1 shows similar findings in analyses stratified by age-group.”*

**4.** An early study with similar design ([https://clicktime.symantec.com/38Wm47moL8c29FYaSHAfHRS6H2?u=https%3A%2F%2Fwww.sciencedirect.com%2Fscience%2Farticle%2Fpii%2F0895435696001953](https://courriel-bsr.aphp.fr/owa/redir.aspx?C=l8VHSwI52g2cQHXdjXjQBWx0NKSWfZ3EC07CPZEXzvNUGSxSTbXXCA..&URL=https%3a%2f%2fclicktime.symantec.com%2f38Wm47moL8c29FYaSHAfHRS6H2%3fu%3dhttps%253A%252F%252Fwww.sciencedirect.com%252Fscience%252Farticle%252Fpii%252F0895435696001953)) showed similar results but looked at the sensitivity and specificity of E4 genotypes and found that the sensitivity was 0.52 /specificity was 0.74 for heterozygotes and homozygotes 0.23 sensitivity/0.99 specificity. The authors concluded that it was significant risk factors but as a diagnostic marker for AD would miss many true cases and misclassify many normal as AD. Can the authors show what the sensitivity and specificity of the biomarker is in their sample and whether or not they believe it has additional added value over and beyond the clinical assessment. Ideally you want to perform a ROC analyses but understand this may be not in the scope of the study analysis.

**OUR RESPONSE:** Thank you, we have added this interesting reference to the revised manuscript. We also report the sensitivity and specificity of *APOE* ε4 to discriminate CSF AD cases from population controls: respectively 56% and 78% for heterozygotes and 31% and 98% for homozygotes, corresponding to an area under ROC curve (AUC) of 0.70 (0.69-0.72, p<0.001). We performed a ROC curve analysis as a function of age that is now shown in Figure 5 in the revised manuscript. The results are described as follows:

**Page 21, line 383-390:**
*“Sensitivity and specificity of APOE ε4 to discriminate CSF AD from population controls was respectively 56% and 78% for heterozygotes and 31% and 98% for homozygotes, corresponding to an area under ROC curve (AUC) of 0.70 (0.69-0.72, p<0.001); similar results were found when comparing CSF AD cases to CSF controls (AUC = 0.70 (0.69-0.73), p<0.001). The ability of APOE ε4 to discriminate AD from controls differed as a function of age (Figure 5), and was greatest at the age of 65-69 years old (AUC=0.78 (0.75-0.80), p<0.001) and poorer before 55 years old (AUC=0.55 (0.47-0.62), p=0.32) and after 85 years old (AUC=0.61 (0.56-0.67), p=0.001).”*

**Figure.** Analysis of Area Under ROC Curve in function of age, for at least one *APOE* ε4 carrying versus none to discriminate CSF AD from population controls.

**5.** Apart from these issues above, the study reveals that the effect sizes were similar but perhaps there conclusion from their crude ORs that the associations between APOE genotypes and AD is much greater than previously reported may actually decrease when consideration of better control selection and covariate adjustment is taken into account. It would be advisable for the authors to consider these issues prior to consideration for publication.

**OUR RESPONSE:** We thank the reviewer again for raising this point. In the revised manuscript we have adjusted for sex, education, and cardiovascular risk factors (please see response to comment 3 above). We show both the unadjusted and adjusted associations in Table 3. We did not adjust for age in these analyses due to evidence of strong effect modification by age; multivariable analysis stratified by age is presented in supplementary Figure 1. Adjustment for cardiovascular risk factors did not modify the strength of the associations between *APOE* genotypes and AD.

**REVIEWER 2**

In this work Saddiki and colleagues aimed to assess the association between AD and APOE genotype in groups of patients based on age-specific increments. In this confirmatory study the studied subjects were included from 9 different European centers, and the ADNI. The rationale for the study is based on previous studies assessing associations between APOE genotype and AD in patients diagnosed based on clinical criteria rather than evidence of AD pathology as assessed by available biomarkers - hence the authors speculated that the previously reported strong association between APOE and AD is an underestimation due to imprecise clinical diagnosis. Results are in line with previous studies reporting a strong association between APOE4 and AD and which is modified by age - less pronounced effect in younger and older individuals. The main take-home is that the association between AD and a homozygous APOE4 genotype is stronger than that reported in previous studies which were based on clinical AD diagnosis without the use of CSF biomarkers. This is an interesting study which is largely confirming previous studies hence novelty is limited yet the take-home message is of importance.

**OUR RESPONSE:** We thank the reviewer for a nice summary of our paper.

This reviewer has several major concerns which need to be addressed before this work can be accepted for publication:
Major:
**1.** The studied subjects were assessed in various different memory clinics which according to the supplementary information used locally-defined CSF biomarker cut-offs which at least for Abeta varied dramatically. Given the variation in AD risk between subjects in different geographical locations (most probably due to impact of environment factors like diet, sun exposure etc) the authors are asked to include a table specifying the number of subjects included from each European center so that the effect of each location on the results can be assessed. This is important especially since the results may be biased by results from specific populations in which amyloid-beta pathology (which is driven mainly by presence of the APOE4 allele both in patients and controls) was more/less pronounced as assessed by CSF AD biomarkers.

**OUR RESPONSE:** Thank you for this excellent suggestion. The number of subjects (CSF AD cases and CSF controls) included from each European center has now been added as Supplementary Table 1 in the revised manuscript. We agree with the reviewer that intersite variability may be an issue when considering CSF biomarkers of AD. To explore further the heterogeneity of the association between centers, we undertook stratified analyses and calculated the I^2^ statistic. The result of this analysis is presented in the Figure below (new Figure 6 in the revised manuscript).

**Figure**. Association between *APOE* ε4 carrying and AD stratified by center and heterogeneity of the study, using general population group as controls.

**ACTION :**

- Page 9, line 183-185:
  “*The number of patients included from each center as well as center specific cut-offs of the CSF biomarkers are presented in Supplementary table 1.”*

The following text has been has been added to the revised manuscript (Page 22, line 394-397):
*“We examined the heterogeneity in associations of one or more APOE ε4 with AD across the different memory centers using population controls as the comparison group. These results are shown in Figure 6. The I2 statistic was 58%, corresponding to some heterogeneity between centers.”*

**2.** Subjects from one of the centers lacked CSF p-tau and amyloid-beta40, the authors need to explain how this affected the A/T/N classification - if incomplete data this reviewer suggests to remove the subjects from that center from the study unless there is a strong rationale to as why to keep them in.

**OUR RESPONSE:** Measurement of phosphorylated tau was missing for one of the center (Gothenburg, n=159 AD cases). We choose to use assessment of Tau as a surrogate marker of phosphorylated tau for this center. Indeed, both variables were highly correlated (Spearman correlation coefficient = 0.84, 95%CI: 0.83-0.85) and the kappa coefficient of agreement between the 2 variables was substantial (kappa = 0.67). We now checked in sensitivity analysis that exclusion of this center has no impact on the results, as presented in the Table below and also included as Supplementary Table 2 in the revised manuscript. The results were reported as follows in the manuscript (Page 17, line 337-339):
*“Using CSF controls instead of population controls yielded similar findings (Table 2), as well as the exclusion of the center with missing data for CSF phosphorylated tau (Supplementary Table 2).”*

**Table.** The Odds ratios of AD in CSF AD cases compared to population controls, before and after exclusion of the center with missing CSF phosphorylated tau.

|  | All centers | |  | Without Gothenburg | |
| --- | --- | --- | --- | --- | --- |
| *APOE* genotype | OR (95% CI) | p-value |  | OR (95% CI) | p-value |
| 0 ε4 | 1 (Ref) | . |  | 1 (Ref) | . |
| ≥ 1 ε4 | 5.9 (5.3 - 6.6) | <0.001 |  | 5.6 (5.0 - 6.3) | <0.001 |
|  |  |  |  |  |  |
| 0 ε4 | 1 (Ref) | . |  | 1 (Ref) | . |
| 1 ε4 | 4.6 (4.1 - 5.2) | <0.001 |  | 4.5 (3.9 - 5.0) | <0.001 |
| 2 ε4 | 25.3 (20.4 - 31.2) | <0.001 |  | 23.6 (19.0 - 29.4) | <0.001 |
|  |  |  |  |  |  |
| ε2/ε2, ε2/ε3 | 0.68 (0.53 - 0.88) | 0.003 |  | 0.62 (0.47 - 0.82) | 0.001 |
| ε3/ε3 | 1 (Ref) | . |  | 1 (Ref) | . |
| ε2/ε4 | 2.9 (2.0 - 4.0) | <0.001 |  | 2.6 (1.8 - 3.7) | <0.001 |
| ε3/ε4 | 4.5 (4.0 - 5.1) | <0.001 |  | 4.3 (3.8 - 4.9) | <0.001 |
| ε4/ε4 | 23.9 (19.3 - 29.6) | <0.001 |  | 22.1 (17.7 - 27.6) | <0.001 |
| Abbreviations: AD. Alzheimer’s disease; *APOE*. Apolipoprotein E; CSF. Cerebrospinal Fluid; OR. odds ratio; CI. confidence intervals. | | | | | |

**3.** The authors should address the p-tau cut-off employed in Antwerpen if it is not a typographical error
**OUR RESPONSE:** We apologize; it was indeed a typographical error that has now been corrected in the manuscript (Supplementary Table 1).

**4.** More information about the controls should be provided. It is widely known that many cognitive controls exhibit altered CSF AD biomarkers with no impact on clinical parameters - the authors need to discuss this aspect.

**OUR RESPONSE:** We indeed don’t know the A/T/N status for the “population controls”; we agree with the reviewer that some cognitive controls may be clinically asymptomatic but have altered CSF AD biomarkers. In order to address this issue, we excluded controls who develop incident dementia during the longitudinal follow-up in these cohort studies. This information has now been included in the revised manuscript, as follows (Page 10, line 197-200):

*“We excluded prevalent cases of dementia at measurement of APOE genotype and incident cases over the subsequent follow-up: 20 years in the Whitehall II study (168 incident cases) and 8 years in the 3-City study (182 prevalent cases and 220 incident cases).”*

In order to further address this issue we used a second population of controls composed of patients coming from the same memory clinics but with normal values for CSF Aβ42, CSF Tau and CSF phosphorylated tau. The proportion of *APOE* ε4 was similar in the 2 populations of controls (22.6% and 21.3%, p=0.41), and logistic regression analysis led to similar results using the both populations as controls. This is discussed in the Discussion section as follows (Page 25, line 436-440):

*“As this control group may not have clinical dementia but may be positive on AD biomarkers, we also considered a second group of controls drawn from the same memory clinics as the AD cases. This group of controls had a normal CSF AD biomarker profile and results using these 2 independent sets of controls were similar, suggesting that our findings are robust.”*

**5.** Included subjects are described as Caucasian, the authors need to state whether ethnicity was self-reported by the study subjects or assessed by the examining physician only by the appearance of skin color. By tradition ethnicity has not been routinely logged in some European countries (contrary to for example the USA) - since the authors explicitly state that the study was performed on Caucasians they need to comment on this assessment.

**OUR RESPONSE:** When constituting the data used for this work, investigators from the participating European centers were asked to provide data on Caucasian patients, with available data for CSF biomarkers of AD and *APOE* genotype. Ethnicity was assessed either by the examining physician, mainly on patient’s interview and examination, or by self-report, following local practices which may differ among participating centers. In order to match the cases, only Caucasian controls were selected.

**6.** The authors do not give a proper rationale to as why non-Caucasians were excluded from the study.

**OUR RESPONSE:** Several previous studies have reported that the APOE allele distribution may depend on ethnicity, with higher ε4 frequencies being more common close to the equator [2]. This might introduce confounding bias in gene-association studies. Therefore, we choose to restrict our analysis to Caucasian subjects, and it was asked to the investigators of the participating centers to share data only on Caucasian participants. We now discuss this point on the Limitations section of the Manuscript. Page 28, Line 510-514:
*“Finally, these analyses were restricted to Caucasian populations as the APOE ε4 allele frequency as well of its association with AD risk may be strongly influenced by ethnicity and geographic areas [44,45]. Further research using different population settings is needed to better understand these complex associations.”*


Minor:
**1.** The authors should explain the A/T/N classification abbreviation in the abstract.

**OUR RESPONSE:** We have now added details to explain the A/T/N classification abbreviation in the Abstract. Page 3, Line 67-69:
*“…using the A/T/N classification, relying on the cerebrospinal fluid (CSF) levels of beta-amyloid peptide (A, beta-amyloid deposition), phosphorylated tau (T, pathologic tau) and total tau (N, neurodegeneration) to identify AD patients.”*

**2.** References are lacking to the methodologies used to assess APOE genotype (M&M).

**OUR RESPONSE:** We have now added some references for assessment of APOE genotype (reference 21 to 24).

**3.** The authors need to explain the term 'CSF AD cases'.  **OUR RESPONSE:** We have now explicated the terms ‘CSF AD cases’ and ‘CSF controls’.

Page 9 Line 174-175:
*“…and had a positive biomarkers profiles according to the A/T/N classification and are referred to as “CSF AD cases” in this study.”*

Page 10 Line 208-210:
*“We also used a second set of controls, identified as “CSF controls”, consisting of Caucasian individuals recruited at the memory centers with normal values for all CSF biomarkers...”*

**4.** There are several language errors (typos) which need to be corrected.
**OUR RESPONSE:** We apologize for these errors, the revised manuscript has now been read carefully by a native English speaker (A.S.-M.).

**5.** The statistical method used to assess group differences should be described in the text and figure legends.
**OUR RESPONSE:** We have now described the statistical methods used in Table 1 (quote a and b), Table 2, Table 3, and legend of Figure 4.

**REVIEWER 3**

In this manuscript Saddiki and colleagues use the rigorous A/T/N framework to re-evaluate risk of AD and APOE genotype. They use a case control design (1,599 cases and 801 controls and 11,724 controls) and calculated odds ratios and population attributable fraction (PAF) for AD association with APOE genotypes and age. They report OR of having AD is 4.6 with one E4 copy and 25.4 with two copies.  They report highest PAF (69.7) between ages 65-70, and significantly weaker PAF at the tails of the age distributions. They find a modest effect of sex with females showing a stronger effect of E4 on AD risk. They conclude that E4 confers even greater risk that previously appreciated in a cohort rigorously defined using A/T/N biomarker criteria . This is a nice study in which the authors make a strong case for the use of the A/T/N framework in order to reduce misclassification noise from clinical diagnosis.  They show that APOE effects on AD risk may be stronger than previously known, vary across aging, and disproportionately affect women.  This is an important study and substantially contributes to our understanding of the two most well-known risk factors for AD- age and APOE. The strengths of the study include biomarker-based diagnosis rather than clinical diagnosis for the primary contrasts (CSF AD vs CSF controls) and the relatively large sample size of all participant groups.  The comparison of the CSF AD and the population control cohort is really an extension of the CSF AD vs CSF control group since the population cohort was not defined using the A/T/N criteria.  However, this is not really a weakness or significant limitation, but inferences about the CSF AD vs population controls should be made with this caveat.  A more concerning limitation is the exclusion of non-Caucasian participants.  The risk for AD is different for Caucasian and non-Caucasians and this is something that can be addressed in future studies.

**OUR RESPONSE:** We thank the reviewer for this nice summary of our paper.

I have a few additional comments:

**1.** Because participants were all Caucasian, other risk factors for AD which are over represented in non-Caucasian populations including vascular and metabolic factors may be under represented here. APOE risk for AD may be overstated in this study because these other risk factors were underrepresented. The authors should state this as a limitation of the study, or alternately qualify all of the findings as applying to Caucasians.

**OUR RESPONSE:** This is an important point, also mentioned by Reviewer 2. As the frequency of *APOE* ε4 and its association with AD risk varies with ethnicity, we chose in the present study to restrict our analysis to the Caucasian population. We have now added this point to the Limitations paragraph of the discussion. Page 28, Line 510-514:

*“Finally, these analyses were restricted to Caucasian populations as the APOE ε4 allele frequency as well of its association with AD risk may be strongly influenced by ethnicity and geographic areas [44,45]. Further research using different population settings is needed to better understand these complex associations.”*

**2.** Its misleading to call the comparison between the CSF AD and CSF controls "secondary analyses" (line 166-168.  The "cleaner" control group here is the CSF controls because they were identified by the more rigorous criteria (A/T/N). Further, its not entirely clear how the CSF controls were recruited and under what conditions. The authors state they came from the memory centers (line 168) but other than stating they were required to have normal A/T/N values its not clear who these participants were. Were non-Caucasian excluded from this group? Were they spouses or siblings of the CSF AD participants?   A little more detail on how these CSF controls were included in the study would be helpful to understand whether they are comparable to the population control group.

**OUR RESPONSE:** We agree, the revised manuscript refers to two sets of controls. The “CSF controls” were all Caucasian, as each center was asked to provide data on Caucasian subjects, and we excluded non-Caucasian subjects from ADNI. These controls were mainly patients assessed for cognitive disorders other than Alzheimer’s disease or normal cognitive subjects coming from ongoing research protocols from the different sites, we unfortunately don’t have information on their diagnosis.

The following text has been added in the Introduction to describe two sets of controls: (Page 8 Lines 161-163):
*“We also considered a second independent control group, recruited from subjects seen at the same memory clinics as the AD cases, who were characterized by a normal profile of CSF AD biomarkers.”*

We also removed the notion of “secondary analysis” and added details on the composition of the “CSF controls” population. Page 10 Lines 208-212:
*“We also used a second set of controls, identified as “CSF controls”, consisting of Caucasian individuals recruited at the memory centers with normal values for all CSF biomarkers (Aβ42, Tau, phosphorylated Tau). This population consisted mainly of patients assessed for cognitive disorders other than AD and/or persons referred to the memory clinic who turned out not have AD.”*

We are reassured by the fact that the 2 sets of controls in our study had very similar *APOE* ε4 distributions, as shown in the Figure 2 of the manuscript.

**3.** The authors might consider minimizing the notion that they "…ensured that the controls didn't include very early stage of dementia" (lines 347-349). To be fair, they did exclude prevalent and incident AD in their population controls through clinical evaluations which does minimize misclassification, but a major premise of this study is that biomarker diagnosis is superior to clinical diagnosis through reducing misclassification bias. Only the 801 CSF control participants met this scrutiny, the 11,724 population controls did not.

**OUR RESPONSE:** We agree with the Reviewer and have tempered this part of the Discussion. Page 25, Lines 436-440:
*“As this control group may not have clinical dementia but may be positive on AD biomarkers, we also considered a second group of controls drawn from the same memory clinics as the AD cases. This group of controls had a normal CSF AD biomarker profile and results using these 2 independent sets of controls were similar, suggesting that our findings are robust.”*

**5.** Table 1 presents a single p value for what is presumably a students t-test for the continuous variables age and MMSE, but there are three groups (CSF AD, CSF controls, population controls). The authors need to clarify which comparison the p value reflects or provide p values for all group comparisons.

**OUR RESPONSE:** We have now clarified this point in Table 1, by presenting the p-values for 2 comparisons: CSF AD vs population controls and CSF AD vs CSF controls, as described in the Methods section.

**6.** Sentences on Lines 123-125 and 349-352 are awkwardly written.

**OUR RESPONSE:** We have rewritten the first sentence and removed the second one.
Page 8 Line 156-159: *“Contrary to previous case-controls studies in which controls may have also included persons at a prodromal stage of AD, we chose a first control group drawn from two large European longitudinal cohorts, the Whitehall II Study [19] and Three-City Study [20] where prevalent and incident cases of dementia over the follow-up were excluded.”*

**References**

1. Hennessy S, Bilker WB, Berlin JA, Strom BL Factors influencing the optimal control-to-case ratio in matched case-control studies. Am J Epidemiol. 1999; 149 (2): 195-197. 10.1093/oxfordjournals.aje.a009786. PMID: 9921965

2. Kern S, Mehlig K, Kern J, Zetterberg H, Thelle D, Skoog I, et al. The distribution of apolipoprotein E genotype over the adult lifespan and in relation to country of birth. Am J Epidemiol. 2015; 181 (3): 214-217. 10.1093/aje/kwu442. PMID: 25609095
