# Supplementary material for: Age and the association between apolipoprotein E genotype and Alzheimer disease: A cerebrospinal fluid biomarker–based case–control study
Source: PLoS Med. 2020 Aug 20;17(8):e1003289. doi: 10.1371/journal.pmed.1003289 (PMC7446786; doi:10.1371/journal.pmed.1003289)
Supplement: Attachment — Submitted filename: Response to reviewers.docx [file pmed.1003289.s008.docx]

**EDITORS**

1. Title: Please include the study population in the title.

**OUR RESPONSE**: Thank you, we have modified the title as follows:

*“Age and the association between Apolipoprotein E genotype and Alzheimer’s disease: a cerebrospinal fluid biomarker-based case-control study on white older adults.”*

2. Abstract (and throughout): Please replace "Caucasian" with "white" throughout the paper.

**OUR RESPONSE:** Done, thank you.

3. Table 1: Please define abbreviation for MMSE in the legend.

**OUR RESPONSE:** Done, thank you.

4. S1 Text: Thank you for including your analysis plan. You note that: “The analyses undertaken were proposed as an MSc internship project advertised via various university channels in November 2018.” Is there any version of the document that incorporates this explanation, or the date, to note that these analyses were pre-specified?

**OUR RESPONSE:** We have updated the “supplementary S1 text” to specify the date and the website where the MSc internship project was advertised:

“This project has been posted on the ISPED website (http://www.isped.u-bordeaux.fr/ISPED/On-recrute), Institut de santé publique, d'épidémiologie et de développement, Unersity of Bordeaux, France, on November 11^th^ 2018 (N° 2018-5380).”

**REVIEWER 2**

The authors have nicely addressed my comments and those of the other reviewers. I have no further concerns.

**OUR RESPONSE:** Thank you.

**REVIEWER 3**

In this revised version of their manuscript, Saddiki and colleagues have made changes that address most of the reviewers concerns. I think this is a strong study which will contribute to the field. The major limitation in my mind is still the restriction to Caucasian participants. Future studies will need to address this finding in more diverse populations.

**OUR RESPONSE:** We agree; there is emerging evidence of racial/ethnic differences in the distribution of APOE ε4 and its association with AD. It would indeed be important to examine this association in diverse populations but unfortunately such data do not exist at the present time.

**REVIEWER 4**

1. I confine my remarks to statistical aspects of this paper. The general approach is fine. I do have a couple of questions and issues to resolve before I can recommend publication. My main question is why the authors chose to add covariates rather than do some form of matching (either by particular values of variables or by propensity score). While it isn't necessarily wrong to use covariates, I think matching is more common.

**OUR RESPONSE:**

Thank you for raising this point, we agree with the reviewer that there are many methods to control for confounding. Several recent papers show that standard multivariable and propensity-score based methods of adjusting for covariates in observational studies yields similar effect estimates (Fox et al., Plos One 2017; Elze et al., J Am Coll Cardiol. 2017). Our view is that propensity-score based methods may be less adapted for a genetic association study like ours when measures such as dyslipidemia may be on the causal pathway. As previously suggested by Reviewer 1, we undertook further analysis to compare the multivariable model that we used to a matched case-control analysis design, by matching on age and sex each AD case to 3 controls, results are presented in the Table below. Both approaches led to very similar findings. Given these results, we feel that there is unlikely to be major bias in the use of multivariable adjustment approach used in our study.

**Table.** Association between APOE genotype and AD : age and sex adjusted model versus age and sex matched case-control study.

|  | **Age and sex adjusted model** | |  | **Matched case-control study^a^** | |
| --- | --- | --- | --- | --- | --- |
| *APOE* genotype | OR (95% CI) | p-value |  | OR (95% CI) | p-value |
| 0 ε4 | 1 (Ref) | . |  | 1 (Ref) | . |
| ≥ 1 ε4 | 6.6 (5.8 - 7.6) | <0.001 |  | 6.8 (6.0 - 7.7) | <0.001 |
|  |  |  |  |  |  |
| 0 ε4 | 1 (Ref) | . |  | 1 (Ref) | . |
| 1 ε4 | 5.2 (4.5 - 6.0) | <0.001 |  | 5.3 (4.6 - 6) | <0.001 |
| 2 ε4 | 33.7 (25.3 - 44.8) | <0.001 |  | 33.9 (24.8 - 46.3) | <0.001 |
|  |  |  |  |  |  |
| ε2/ε2, ε2/ε3 | 0.54 (0.39 - 0.74) | <0.001 |  | 0.71 (0.55 - 0.93) | 0.012 |
| ε3/ε3 | 1 (Ref) | . |  | 1 (Ref) | . |
| ε2/ε4 | 3.3 (2.1 - 5.1) | <0.001 |  | 3.6 (2.4 - 5.2) | <0.001 |
| ε3/ε4 | 4.9 (4.2 - 5.8) | <0.001 |  | 5.2 (4.5 - 5.9) | <0.001 |
| ε4/ε4 | 31.0 (23.3 - 41.3) | <0.001 |  | 32.3 (23.6 - 44.2) | <0.001 |
| ^a^Each AD cases were matched with 3 controls on age and sex. | | | | | |

References

- Fox GJ, Benedetti A, Mitnick CD, Pai M, Menzies D; Collaborative Group for Meta-Analysis of Individual Patient Data in MDR-TB. Propensity Score-Based Approaches to Confounding by Indication in Individual Patient Data Meta-Analysis: Non-Standardized Treatment for Multidrug Resistant Tuberculosis. PLoS One. 2016 Mar 29;11(3):e0151724. doi: 10.1371/journal.pone.0151724. EFS
- Elze MC, Gregson J, Baber U, Williamson E, Sartori S, Mehran R, Nichols M, Stone GW, Pocock SJ. Comparison of Propensity Score Methods and Covariate Adjustment: Evaluation in 4 Cardiovascular Studies. J Am Coll Cardiol. 2017 Jan 24;69(3):345-357.

2. Lines 274-279 I am confused as to why both these were done - I would not have done the analysis with categorized age. Categorizing independent variables is almost always a mistake. But then the authors also did he analysis with age as continuous and added a quadratic term. So, what was the first analysis for? (Also, the authors could explore use of a spline method and see if it does much better than just the quardratic).

**OUR RESPONSE:** Apologies for not being clear. The independent variable in these analyses is APOE and the outcome is AD. Given the large sample size, our objective was to examine whether the association between APOE and AD differs as a function of age. Thus, we first examined this association in analysis stratified by 5-years age group. Then we tested for the shape of the association using an interaction between APOE and age^2^. We have clarified our approach in the revised the Statistical methods section of the manuscript (page 13, lines 275-279):

*“Cumulative percentage of CSF AD cases as a function of age (in 5-year age-groups) were plotted according to the APOE ε4 status (0, 1, 2 alleles); median age in the three groups were compared using the non-parametric Brown-Mood median test. We then used logistic regression to estimate the odds ratio (OR) of AD according to APOE genotype, comparing the total population of CSF determined AD cases to population controls first and then to CSF controls. The role of age in modifying the association between APOE genotype and AD was examined in analyses stratified by 5 year age-group, ranging from <55 years to >85 years. The ε3/ε3 group was the reference and analysis undertaken in groups using ε4 status. The non-linearity of associations with AD as a function of age was tested using an interaction term between age^2^ and APOE.”*

3. Figs. 2 and 3 - Stacked bar charts are not a good method (see the work of William S. Cleveland) It would be better to use a line graph with 3 lines in each panel of fig 2 and 2 lines in fig. 3.

**OUR RESPONSE:** We thank the reviewer, the revised manuscript includes the changes recommended by the reviewer (Figures 2 and 3 as line graphs).
