# Supplementary material for: Age and the association between apolipoprotein E genotype and Alzheimer disease: A cerebrospinal fluid biomarker–based case–control study
Source: PLoS Med. 2020 Aug 20;17(8):e1003289. doi: 10.1371/journal.pmed.1003289 (PMC7446786; doi:10.1371/journal.pmed.1003289)
Supplement: Attachment — Submitted filename: Response to reviewers.docx [file pmed.1003289.s009.docx]

**EDITORS**

1. Title: We appreciate your willingness to revise your title; please remove “on white older adults” from the title.

**OUR RESPONSE:** Done, thank you.

2. Data availability statement: The Data Availability Statement (DAS) requires revision. For each data source used in your study: if the data are owned by a third party but freely available upon request, please note this and state the owner of the data set and contact information for data requests (web or email address). Note that a study author cannot be the contact person for the data.

Please revise your statement to include the relevant contact information for requesting data access for the ADNI Study, 3-City Study and Whitehall II Study, and please note that the contact cannot be one of the authors of the manuscript.

Please also update the link for the Whitehall-II study, as it does not seem to work: <https://clicktime.symantec.com/3WPhPkVo9AWyX5S9hS25VJo6H2?u=https%3A%2F%2Fwww.ucl.ac.uk%2Fepidemiology-health-care%2Fresearch%2Fepidemiology-and-publichealth%2Fresearch%2Fwhitehall-ii>

**OUR RESPONSE:** Done, thank you.

3. Abstract: Line 84: Please replace "subject" with participant, patient, individual, or person.

**OUR RESPONSE:** Done, thank you.

4. Abstract: Conclusions: Please revise the first sentence to better reflect on the study’s findings: “In this study, we found that AD was associated with APOE ε4 carrier status, with a higher OR than previously reported for associations between AD diagnosis and biomarker status.” or similar.

**OUR RESPONSE:** Done, thank you.

5. Author summary: “What did the researchers do and find?”: We suggest removing the word “strongly” from the third bullet point, and replacing with “significantly”

**OUR RESPONSE:** Done, thank you.

6. Author summary: “What do these findings mean?”: We suggest revising the first bullet point to: “Incorporating biomarkers for diagnosis of AD identified an association with APOE4 that is apparently greater than has been previously reported using clinical diagnosis of the disease.” or similar, as the objective of the study does not seem to be to compare associations between this and other studies.

**OUR RESPONSE:** Done, thank you.

7. Introduction: Line 163: Please replace "subject" with participant, patient, individual, or person.

**OUR RESPONSE:** Done, thank you.

8. Methods: Line 194: “Term” should be “terms”

**OUR RESPONSE:** Done, thank you.

9. Results: Line 317: Please revise this sentence to “The proportion of APOE ε4 carriers…”

**OUR RESPONSE:** Done, thank you.

10. Results: Line 366: Please revise this sentence to “There was no evidence that the age-related association of APOE ε4 differed by sex…”

**OUR RESPONSE:** Done, thank you.

11. Discussion: Line 434 and 438: Please spell out the word “two” rather than “2” here.

**OUR RESPONSE:** Done, thank you.

12. Discussion: Line 447: Should “biomarkers” be “biomarker” here?

**OUR RESPONSE:** We agree and have changed it accordingly.

13. Discussion: Line 452: Please revise to avoid implying causality“...AD is strongly dependent on age at diagnosis.” to “...AD is mediated by age at diagnosis.” In addition, would it be more appropriate to delete “at diagnosis” because your study only indirectly associated age at CSF study with time of diagnosis?

**OUR RESPONSE:** We agree and have changed this sentence accordingly.

14. Discussion: Line 457 and 460: Why is “AD” in quotations here?  Please remove the quotes if not necessary.

**OUR RESPONSE:** Done, thank you.

15. Discussion: Conclusions paragraph:  Please revise the first sentence of the conclusions paragraph to better reflect your study’s findings.  “This study supports an association between APOE ε4 and the risk of AD evaluated using biomarkers of AD neuropathology, and the results suggest that the strength of this association varies by age.”

**OUR RESPONSE:** Done, thank you.

16. Figure 1: Please change “allele” to “alleles”.  Please change the X axis to read “Age at CSF testing” or similar.  In the legend, please explain the dashed horizontal line at 50.

**OUR RESPONSE:** Done, thank you.

17. Figure 2: Please provide an X axis label.

**OUR RESPONSE:** Done, thank you.

18. Figure 6: Please include an X axis label indicating the values are OR and 95% CIs.

**OUR RESPONSE:** Done, thank you.

19. Supporting information Figure 1: In the title, there should be a space between CSF and AD.

**OUR RESPONSE:** Done, thank you.

20. Supplementary table 1: Is it possible to elaborate in the legend, or use a more specific term than “cut-off” as it isn’t quite clear exactly what it means.

**OUR RESPONSE:** Done, thank you.
